# Using Embedded Temperature Sensors to Detect Package Tampering †

**DOI:** 10.3390/s25144250

**Published:** 2025-07-08

**Authors:** Geoffrey Chancel, Julien Toulemont, Frederick Mailly, Philippe Maurine, Pascal Nouet

**Affiliations:** 1 LIRMM, University of Montpellier, 34095 Montpellier, France; gchancel@lirmm.fr (G.C.); frederick.mailly@umontpellier.fr (F.M.); pascal.nouet@umontpellier.fr (P.N.); 2 ANSSI, 75700 Paris, France; julien.toulemont@ssi.gouv.fr

**Keywords:** countermeasures, thermal dissipation monitoring, hardware security, fault injection, reverse engineering

## Abstract

Secure integrated circuits are vulnerable to numerous threats and attacks throughout their lifespan. A noticeable group of these threats is semi-invasive attacks that necessitate removing the package, either from the front or the back. To the best of our knowledge, there has been little research focusing on verifying the package integrity. This paper presents an affordable solution for verifying the package integrity at power-up. This solution relies on an indirect and built-in measurement of the IC’s heat dissipation characteristics, taking advantage of the use of thermal sensors embedded in today’s ICs.

## 1. Introduction

Hardware security is now a major focus in the design of integrated circuits. The growth of the Internet of Things and digital services has made hardware security a critical consideration during the IC design process. This involves addressing threats like side-channel attacks, reverse engineering, fault injection, and counterfeiting.

Attacks are typically categorized by their invasiveness. Non-invasive attacks do not require physical contact with the ICs. Invasive attacks necessitate physical contact with or modification of the IC’s active components, sometimes using tools like a focused ion beam. Semi-invasive attacks fall between these categories, requiring only the removal of the metal or plastic package, which can be achieved relatively inexpensively with a cutter or acids, respectively.

Semi-invasive attacks are varied and include techniques such as Laser Fault Injection (LFI) [1,2,3], Body Biasing Injection (BBI) [4,5], Electromagnetic Fault Injection (EMFI) [6,7], and probing attacks [8,9]. BBI uses small metal probes making contact with the silicon substrate, allowing the attacker to inject energy in the IC thanks to voltage spikes. During the energy propagation, internal voltage drops occur, causing transient IC faults. LFI uses a laser beam to create a parasitic current in the polarized PN junction of logic gates. It creates unexpected transient voltages at the logic gates output. If this voltage is sampled by registers, it can become a fault. Both BBI and LFI require the removal of the IC’s backside package: the first one because a physical contact is required, the second one because the laser beam passes through the substrate. EMFI, on the other hand, does not necessitate package tampering, but is way more effective if the package’s frontside is removed beforehand.

To prevent these threats, various embedded countermeasures have been proposed over time (the following list is not exhaustive):

- **Sensor proximity detection:** Refs. [10,11] proposed to detect changes in electromagnetic susceptibility in the immediate vicinity of the IC frontside caused by any tool, but more specifically electromagnetic probes. For this purpose, the authors suggested embedding coils in the IC and monitoring the change in their resonant frequency.
- **Substrate thinning detection:** Substrate thinning is a very common practice to improve the efficiency of Laser Fault Injection (LFI) or Body Biasing Injection (BBI). To avoid substrate thinning, Refs. [12,13] proposed to integrate etched holes in the substrate so that any thinning attempt leads to the destruction of the IC.
- **Laser beam deflection:** To reduce the efficiency of LFI, Ref. [14] proposed to integrate in the substrate micro-mirrors within a random pyramidal shape to reflect the laser beam, thereby reducing energy concentration.
- **Anti-intrusion active shields:** Made of labyrinth metal wire arrays whose electrical characteristics are monitored to detect any physical intrusion through the IC frontside [14], for instance, to prevent probing attacks. Some other approaches using through silicon vias [13] have been proposed to detect intrusions from the backside substrate or to detect if it has been thinned.
- **Detection of internal disturbances:** Several specialized sensors have been proposed to detect disturbances induced by various common fault injection techniques. Most of these sensors detect a sudden unexpected change in internal signals, such as the following:
  - Supply voltage pulse detectors, which are designed to identify surge voltages by measuring sudden and significant changes in the power supply network [11];
  - Light sensors, designed to detect infrared light, specifically to intercept laser beams, and, therefore, to detect LFI attempts [10];
  - Substrate current detectors, used to detect current surges in the substrate, which can be caused by LFI or BBI [3,15];
  - Electromagnetic pulse detectors, designed to detect electromagnetic pulses occurring in the vicinity of the integrated circuit [3,15].

Although effective, these countermeasures often require specialized designs and can be expensive in terms of design effort, area, and power consumption. Certain methods, particularly those involving backside verification such as the ones using Through Silicon Vias or nano-pyramidal etching, are especially costly due to the need for additional manufacturing processes.

Given that secure applications are increasingly embedded in microcontrollers and SoCs within complex packages (acting, among other things, as heatsinks), this paper investigates, in depth, a method introduced in [16] for checking IC package integrity by monitoring the IC’s ability to dissipate heat.

This approach holds promise for cost and potential, as packages are designed to enhance thermal dissipation and modern ICs commonly include temperature sensors.

To assess the practicality of checking IC package integrity by monitoring its thermal dissipation capability, an experimental approach was taken. This involved selecting a standard microcontroller and attempting to implement a package integrity verification protocol using its built-in features.

The remainder of the paper is structured as follows. Section 2 introduces and justifies the choice of the device under test. Section 3 analyzes the device thermal response during power-up, from which a procedure for monitoring its thermal dissipation capability is developed. Section 4 examines the impact of package removal and proposes a protocol for checking package integrity, in addition to its robustness against circumvention techniques. Section 5 discusses the potential of the proposed method, with specific attention paid to the benefits of employing a more precise thermal sensor. Finally, Section 6 concludes the paper.

## 2. The Device Under Test

### 2.1. General Features

Several microcontrollers incorporate temperature sensors, including those from NXP (LPC family) and STMicroelectronics (STM32 family), which are versatile and commonly used across numerous applications. For our experiments, we selected the STM32F439ZGT6 from STMicroelectronics (Grenoble, France) which features moderate power consumption (145 mW at rest). It includes an ARM Cortex-M4 core and offers 256 kB of RAM and 1024 kB of FLASH memory, alongside various peripherals like a cryptographic core and standard buses, making it suitable for diverse applications. Its die area is approximately 5.5 mm × 4.4 mm.

### 2.2. Temperature Sensor

Most importantly, the microcontroller is equipped with a temperature sensor capable of operating between −40 °C and 125 °C, with an accuracy of ±1.5 °C. Temperature data is digitized into 12-bit values via an integrated Analog-to-Digital Converter (ADC).

To account for manufacturing variations, the manufacturer recommends using the temperature sensor for differential measurements instead of absolute temperature readings. However, each sensor undergoes calibration after manufacturing, and two calibration values, designated TS_CAL1 and TS_CAL2, are stored in non-volatile memory. These values can be retrieved and used to correct temperature measurements, as detailed in the IC datasheet and using the following formula:

(1) $$T=\frac{80}{TS_{CAL1}-TS_{CAL2}}\cdot \left(TS-TS_{CAL1}\right)+30$$

where

- *T* the corrected temperature in °C;
- *TS* is the temperature returned by the sensor in °C.

### 2.3. The Package

**Figure 1:**
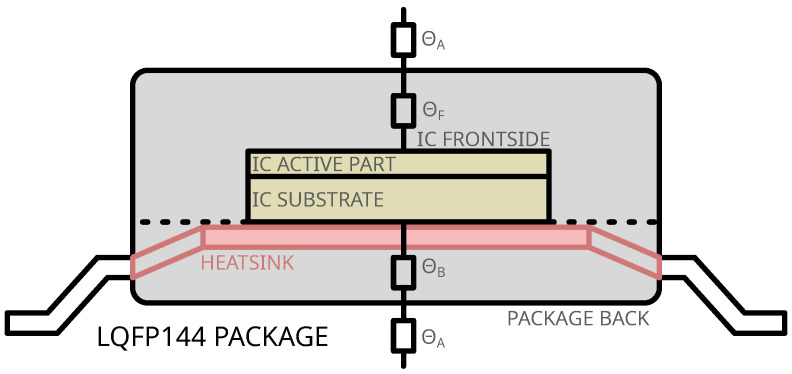
IC package sectional view.

The Device Under Test (DUT) used in this study is packaged in an LQFP-144. In addition to the standard epoxy packaging, a metallic ground plane (and heatsink) is present on the backside of the IC. Figure 1 illustrates the typical layer stack-up of an LQFP-144 package, which, from top to bottom, includes the following layers:

- An epoxy (and more precisely a mold compound [17]) layer between the package frontside and the IC frontside;
- The die with its active components and substrate;
- The internal ground plane, also functioning as a heatsink, in contact with the IC backside;
- A second mold compound layer covering the backside heatsink.

Figure 1 also presents the thermal resistances:

- $\Theta_{A}$: thermal resistance package/air;
- $\Theta_{F}$: frontside thermal resistance (mold compound only);
- $\Theta_{B}$: backside thermal resistance (metal and epoxy).

Given that the backside metal plane is typically grounded, the backside exhibits lower thermal resistance compared to the frontside ($\Theta_{B}<<\Theta_{F}$). Consequently, the removal of the frontside mold compound significantly reduces the thermal resistance $\Theta_{F}$ to a negligible value. In fact, one can consider that only $\Theta_{A}$ remains and the IC directly dissipates part of the heat directly in the ambient air. This leads to a slight decrease of the overall heat dissipation capability because the epoxy resin has high thermal conductivity with respect to air. Conversely, the backside thermal resistance increases significantly after the removal of the backside package, including the metal plane.

Despite the expertise of the company representative that did perform the opening of the ICs considered in the rest of the paper, predicting the thermal behavior after the opening appears very difficult and is also difficult before the opening. Indeed, process variations occur at all manufacturing stages of the IC and of the package, as well as during the opening process, which involves hand manipulations and a kind of learning phase from the expert to finely tune the parameters of the mechanical polishing machine used to create a cavity as well as the deposit of an acid drop. As a first illustration, the process variations in the package manufacturing are, according to the datasheet of the product, in the range of 0.1 mm for all dimensions of the LQFP-144 package. As a second illustration of the magnitude of such variations, the width, length, and depth of the openings (the uncovered part of the substrate) performed on our ICs were measured using a digital microscope. The maximum observed differences, from one circuit to another in the width, length, and depth of the created cavities are about 125 μm, 149 μm, and 153 μm, respectively.

As a result, these random variations render any fine prediction of the thermal behavior of the thermal resistance between the air and the IC a difficult task. We could thus imagine that a package integrity verification solution allowing the detection of package tampering could be hard to bypass.

## 3. Thermal Behavior of the DUT

### 3.1. Preliminary Study of the Thermal Behavior

To establish a methodology for package integrity testing, the thermal behavior of the DUT was analyzed to address the following questions:

- How does removing the front or back of the package alter the thermal behavior of the DUT?
- What time constant characterizes the thermal behavior of the DUT?

To answer these questions, three ICs were subjected to a repeated 320 s sequence. The first IC had its package opened on the frontside, the second had its package opened on the backside, and the third served as a control with its package intact. The first 160 s of this sequence involved allowing the device to remain at rest. Then, a heating phase was applied during the subsequent 160 s. It consisted in writing the data provided by the temperature sensor into the flash memory instead of the RAM, since the flash memory consumes more power than the RAM. This heating phase was used in early experiments to exacerbate the thermal behavior.

**Figure 2:**
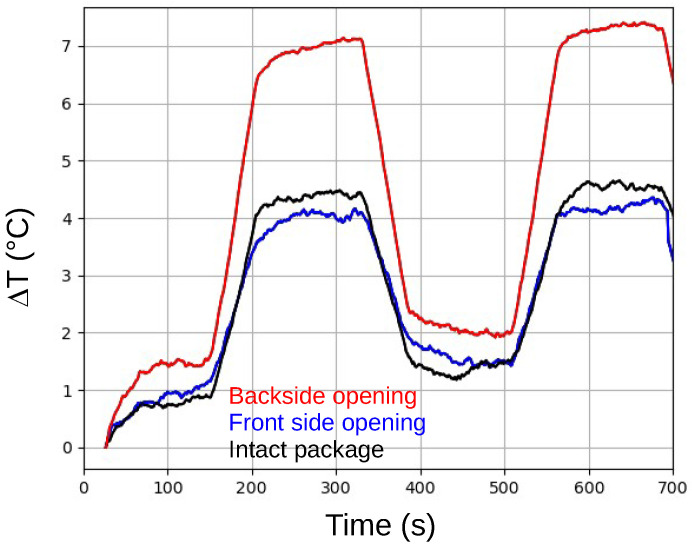
Evolution of the internal temperature difference (ΔT) relative to the temperature at t = 0 s, for the DUT in an intact package (black), a frontside-opened package (blue), and a backside-opened package (red).

Figure 2 displays the evolution of the internal temperature difference (ΔT) for the three ICs, relative to the temperature at t = 0 s. Due to the limited accuracy of the sensor, a moving average was applied to the data to improve readability. As expected, a repetitive pattern with a period of 320 s is visible in all three traces.

An analysis of Figure 2 reveals that the thermal behavior of the IC with an intact package closely resembles that of the IC with the frontside package removed. Given the minimal differences observed, detecting frontside package opening through monitoring the device’s thermal dissipation characteristics appears difficult. Therefore, the subsequent sections of this paper focus on verifying the integrity of the package’s backside.

In contrast, the IC with a backside opening exhibits a more dynamic temperature profile and significantly greater temperature differences compared to the IC with an intact package. This increased difference can likely be attributed to the removal of the heatsink integrated into the package. Regardless of the precise cause, it appears feasible to assess package integrity by monitoring thermal dissipation capabilities.

Figure 2 also shows another important feature of the IC thermal behavior: the later behavior depends on the response time of the internal temperature, which is ultimately quite fast.

### 3.2. Temperature Evolution at IC Power-Up

**Figure 3:**
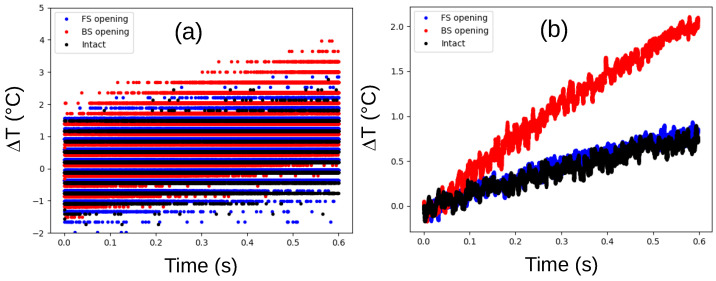
Evolution of the internal temperature difference (ΔT) relative to the temperature at t = 0 s for the DUT with an intact package (black), in a package with an opening on the frontside (blue), and in a package with an opening on the backside (red), immediately after IC power-up. (**a**) Raw measurements provided by the temperature sensor. (**b**) The same measurements after applying a moving average.

Encouraged by the previous observations, we focused on the initial thermal response immediately after powering up the DUT. Figure 3a displays the raw ΔT values during the first 600 milliseconds, revealing a noticeably sharper temperature increase for the IC with the backside opening, even considering the limitations of the sensor’s accuracy. This trend is reinforced by the moving-average data presented in Figure 3b, which demonstrates that the temperature rises more than twice as quickly in the IC with the backside opening compared to the others. These results strongly suggest the feasibility of assessing package backside integrity through monitoring of thermal dissipation.

### 3.3. Checking Package Integrity by Thermal Dissipation Capability at Power-Up

Given the linear trend of ΔT, the temperature difference, and consequently the internal temperature *T*, observed within such a short period after power-up, a simple linear regression can be used to model the internal temperature:

(2) $$T=\beta_{1}\cdot t+\beta_{0}+\epsilon$$

where $\beta_{1}=\frac{cov(T,t)}{V(t)}$ represents the slope of the linear regression, $\beta_{0}$ the y-intercept, and ϵ the modelling error. In the expression for $\beta_{1}$, cov(T,t) stands for the covariance between the temperature values provided by the sensor and the values output by the timer, and V(t) stands for the variance in the values provided by the timer.

The accuracy of this linear model can be assessed, as usual, by considering the coefficient of determination, which is the ratio of the explained variance by the model of the total variance—in this case,

(3) $$R^{2}=\frac{cov^{2}\left(T,t\right)}{V(T)\cdot V(t)}$$

The coefficient of determination, $R^{2}$, ranges from 0 to 1. An $R^{2}$ value of 1 indicates a perfect fit between the model and the observed data, while a value of 0 signifies that the model does not fit the data or that the slope ($\beta_{1}$) is zero.

Within this linear model, $\beta_{1}$ can be considered an indirect measurement of the IC’s thermal dissipation characteristics. Specifically, its value directly reflects the rate of change of the internal temperature sensor and, therefore, the IC’s heat dissipation.

This model facilitates a straightforward method for verifying package integrity during power-up. The approach involves simply confirming that $\beta_{1}$ falls within an acceptable range—for example, $\left[\beta_{1}^{min},\beta_{1}^{max}\right]$—which can be determined during manufacturing and packaging, and is stored in non-volatile memory, similar to TS_CAL1 and TS_CAL2. These values represent unique, per-IC calibration values for the embedded temperature sensor, obtained after manufacturing.

Naturally, variations in both the IC manufacturing and packaging process can influence the $\left[\beta_{1}^{min},\beta_{1}^{max}\right]$ interval. Therefore, this interval should be established by measuring $\beta_{1}$ multiple times for each IC and calculating $\left[\beta_{1}^{min},\beta_{1}^{max}\right]$ as $\left[\bar{\beta_{1}}-3\cdot \sigma_{\beta_{1}},\bar{\beta_{1}}+3\cdot \sigma_{\beta_{1}}\right]$.

It is noteworthy that the coefficient of determination ($R^{2}$) could have been utilized to further enhance the package integrity check by ensuring that $R^{2}$ also falls within an acceptable range. Verifying that $R^{2}$ falls within a defined range, in addition to $\beta_{1}$, would allow us to confirm the linearity of the temperature trend and therefore the detection of rapid temperature changes, forced for instance by an adversary attempting to circumvent the package integrity check by manipulating the $\beta_{1}$ measurement by external means (see Section 4). This particular aspect, however, is not addressed in the present work. The use of a more precise sensor is mandatory. In fact, the sensor embedded in our DUT exhibits a limited accuracy, thereby constraining the range of $R^{2}$ values to a relatively low level.

## 4. Experimental Verification: Soundness and Robustness of the Package Integrity Verification Protocol

Several experiments were conducted to validate the proposed package integrity verification protocol. These experiments aimed not only to verify its correctness but also to assess its robustness under varying IC operating conditions. Additionally, some experiments focused on testing the feasibility of potential bypass solutions to this protection mechanism.

All experiments were performed using six STM32F439ZGT6 devices. To monitor the internal temperature and acquire the $\beta_{1}$ values, the temperature was sampled at a rate of 83,333 samples per second, corresponding to a temperature measurement every 12 µs.

### 4.1. Reliability Assessment of the Package Integrity Verification Protocol

Prior to being sent to a security certification company that specializes in creating package openings, each device underwent characterization and was marked with an identifier (AA, AB, etc.). The method used for the opening of the backside or frontside was the same method that the company used to apply to prepare IC for laser error injections. According to our discussions with company representatives, the opening process consisted of first mechanically polishing the package to remove most of it. This step creates a square cavity into which a small drop of acid is introduced to remove the remaining package. Marking the devices enabled us to track the change in thermal behavior induced by opening the back of each device.

The performed characterization involved measuring the mean ($\bar{\beta_{1}}$) and standard deviation ($\sigma_{\beta_{1}}$) of $\beta_{1}$. The same characterization process was repeated after the DUT’s return.

Table 1 presents the means and standard deviations obtained for each sample, demonstrating the impact of opening the backside package. As anticipated, this action resulted in a significant increase in the $\bar{\beta_{1}}$ value. The observed values of $\bar{\beta_{1}^{\prime }}$ ranged from 1.82 to 5.33 times the original $\bar{\beta_{1}}$ values.

To further validate the soundness of the proposal, we computed the probability (assuming all distributions are normal) of obtaining a $\beta_{1}^{\prime }$ value within the interval $\left[\bar{\beta_{1}}-3\sigma_{\beta_{1}},\bar{\beta_{1}}+3\sigma_{\beta_{1}}\right]$ after backside package opening, effectively bypassing the tamper detection mechanism. The largest probability value observed across the six circuits was $10^{-6}$.

Despite these results, which support the proposed protocol for verifying the integrity of packages, the following can also be observed:

- The $\beta_{1}$ of ICs with an intact package varies from 1.4 °C/s to 3.1 °C/s, highlighting the impact of the manufacturing and packaging process.
- The $\beta_{1}$ increase with backside opening is not uniform. It ranges from 2 °C/s to 6.07 °C/s. This was quite surprising, since the opening mainly removes the heatsink. This suggests that package opening, which involves mechanical and/or chemical processes, in addition to human intervention, is prone to significant variations.

### 4.2. Packaging Integrity Verification Protocol Robustness to Changes in Operating Conditions

The experiments performed to demonstrate the soundness of the test protocol were all conducted in a room with fairly stable operating conditions. Thus, one may wonder if intentional or unintentional variations in the IC operating conditions could cause some false positives or failure to detect package tampering. Therefore, in order to provide answers, additional experiments were conducted.

#### 4.2.1. Robustness to Ambient Temperature Changes

From engineering common sense, one might think that an IC $\beta_{1}$ distribution is independent of the ambient temperature of the room in which it operates.

In fact, $\beta_{1}$ can be viewed as a measure of the IC’s thermal resistance ($\theta_{A}+\theta_{B}$), which is expected to remain stable within a reasonable range of ambient temperatures. To verify this, we measured and recorded $\beta_{1}$ values, as well as the internal temperature values, before and after a measurement of $\beta_{1}$, during five days. The measurements were taken every 30 s. During the acquisition of these values, the ambient temperature around the IC was also measured using an external sensor.

**Figure 4:**
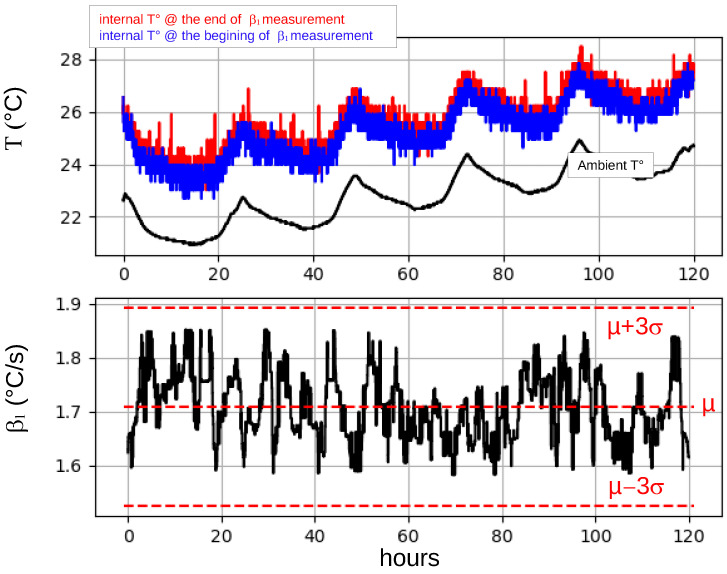
Evolutions, measured during five days, of the ambient temperature (black) and of the temperatures before (blue) and after (red) each $\beta_{1}$ measurement.

The trends observed during these 120 h are shown in Figure 4. The first subplot shows the temperature trends inside the circuit before (in blue) and after (in red) a $\beta_{1}$ measurement. It also shows the temperature trend around the IC. The three monitored temperatures follow exactly the same trend: a periodic variation due to temperature changes during the day and night, and a slow increase in the average temperature by 2 °C between the first and the last day. To support this observation, the correlations between the internal temperatures and the ambient temperature were calculated. They were found to be equal to 0.96 and 0.97, respectively, before and after the $\beta_{1}$ measurement.

The second plot shows the measured $\beta_{1}$ values. Notice that the variations in $\beta_{1}$ do not seem to be correlated with changes in ambient temperature. Furthermore, they remain within the range of $\left[\bar{\beta_{1}}-3\cdot \sigma_{\beta_{1}},\bar{\beta_{1}}+3\cdot \sigma_{\beta_{1}}\right]$. This supports our intuition: $\beta_{1}$ is independent of (or at least weakly dependent on) the ambient temperature.

However, to further support this observation, a one-way analysis of variance [18] was performed between the ambient temperature and the values of $\beta_{1}$. The final value of the F-statistic indicated a weak relationship between the two. In addition, the cluster means indicated that $\beta_{1}$ tended to very slowly decrease as the ambient temperature increased. Therefore, the correlation between the ambient temperature and the values of $\beta_{1}$ was also calculated. A value of −0.22 was found.

A linear regression analysis was also performed and it was found that the value of $\beta_{1}$ very slowly decreases with the ambient temperature at a rate equal to −0.01 per degree.

These results suggest that $\beta_{1}$ is very weakly dependent on the temperature around the DUT. However, considering the limited range (only from 20 °C to 25 °C) of temperatures experienced by the DUT during the five days, we considered these results insufficient. In fact, the temperature range was too narrow and there was no clear evidence that the obtained trends were due to the DUT and not to the temperature sensor used to measure the ambient temperature. Therefore, additional experiments were performed, only involving the DUT, in a climate chamber.

#### 4.2.2. Robustness to Large, Intentional, and Unintentional Ambient Temperature Changes

Experiments in a climate chamber have two interests: the first is to decide if the ambient temperature has a real impact on $\beta_{1}$, and the second is to determine if an adversary aiming to bypass the package integrity verification can control the value of $\beta_{1}$ using a controlled temperature environment.

The first experiment was performed on the same IC (with an intact package) whose thermal behavior, analyzed during five days, is described in the previous paragraphs. Its $\bar{\beta_{1}}$ is equal to 1.7. Twenty new measurements of its $\beta_{1}$ were taken at a controlled temperature equal to 15 °C. Then, twenty more were collected at 45 °C. Taking the trend revealed by the ANOVA and the linear regression as true, we expected a change in $\bar{\beta_{1}}$ equal to −0.3.

**Figure 5:**
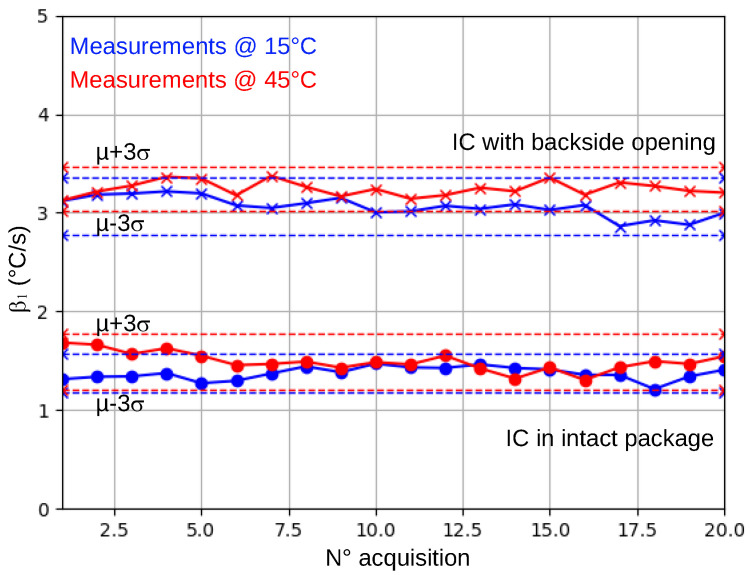
$\beta_{1}$ values for an intact IC and a backside-opened IC at 15 °C and 45 °C ambient temperatures.

Experimental results shown in Figure 5 indicate that the weak trend revealed by statistical means is an artifact or is due to the thermal sensor used to measure the ambient temperature around the DUT. In fact, $\bar{\beta_{1}}$ is the same after a 30 °C change (from 15 °C to 45 °C) in ambient temperature. Furthermore, all the collected values of $\beta_{1}$ remained in $\left[\bar{\beta_{1}}-3\cdot \sigma_{\beta_{1}},\bar{\beta_{1}}+3\cdot \sigma_{\beta_{1}}\right]$. This is a first demonstration of the robustness of the proposed package integrity verification protocol against unintentional or intentional changes in ambient temperature. As a second demonstration, the same experiment was repeated with an IC whose package was opened from the backside. The results obtained, also shown in Figure 5, led us to the same conclusion.

#### 4.2.3. Robustness to Large, Intentional, and Unintentional Supply Voltage Changes

Similar to ambient temperature, the IC supply voltage $V_{DD}$ can be inadvertently altered or intentionally modified to attempt to bypass the package integrity test. Indeed, one could expect a change in the power consumption of the IC, which is proportional to $V_{DD}^{2}$, when lowering the supply voltage.

**Figure 6:**
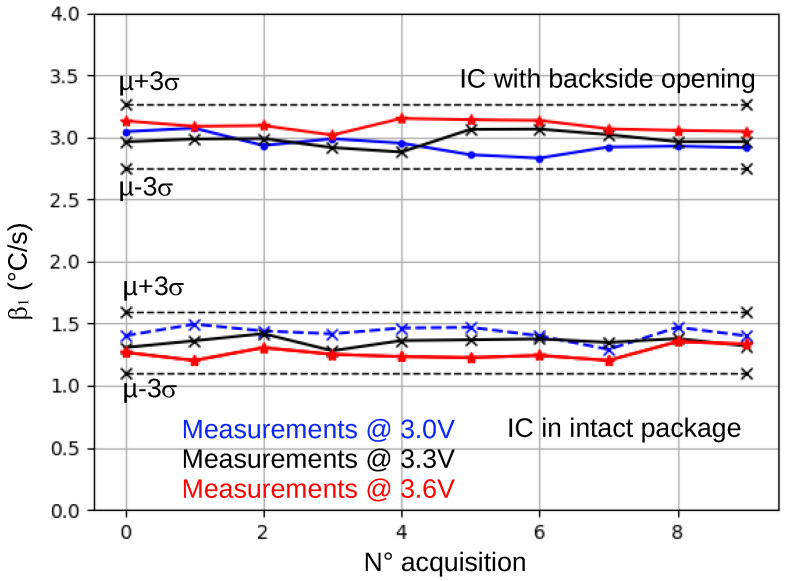
$\beta_{1}$ values for an intact IC and a backside-opened IC, measured for 3 V, 3.3 V, and 3.6 V supply voltage.

Therefore, new $\beta_{1}$ measurements were performed on the same two ICs at different supply voltages: 3.0 V, 3.3 V, and 3.6 V. Results are shown in Figure 6. As for experiments related to large changes in the ambient temperature, all collected $\beta_{1}$ values remained in $\left[\bar{\beta_{1}}-3\cdot \sigma_{\beta_{1}},\bar{\beta_{1}}+3\cdot \sigma_{\beta_{1}}\right]$. This result was expected since the IC contains a voltage regulator that stabilizes the CPU core voltage at 1.2 V. Thus, it cannot be concluded that $\beta_{1}$ is independent of $V_{DD}$. However, it can be concluded that, if the IC includes an efficient voltage regulator, as the STM32F439ZGT6 and many modern microcontrollers do, the package integrity verification protocol is protected against intentional supply voltage changes.

### 4.3. Packaging Integrity Verification Protocol Robustness to Intentionally Forced Thermal Behavior

The previous section focused on the robustness of the packaging integrity verification protocol to unintentional and intentional changes in static operating conditions. This section examines some low-cost solutions to bypass the verification, knowing that there are attacks that can be used to do so, such as fault attacks, especially LFI, which could force a positive comparison of $\beta_{1}$ with the upper and lower bound of the acceptable range.

There are two main solutions to bypass the verification protocol. The first approach is to restore the initial thermal behavior of the IC, for example, by using a removable heat sink that is removed once the IC has completed the package integrity verification process. The second one is to force a temporary thermal behavior so that to mislead the verification protocol, that is to say, the linear regression.

#### 4.3.1. Removable Heatsink

**Figure 7:**
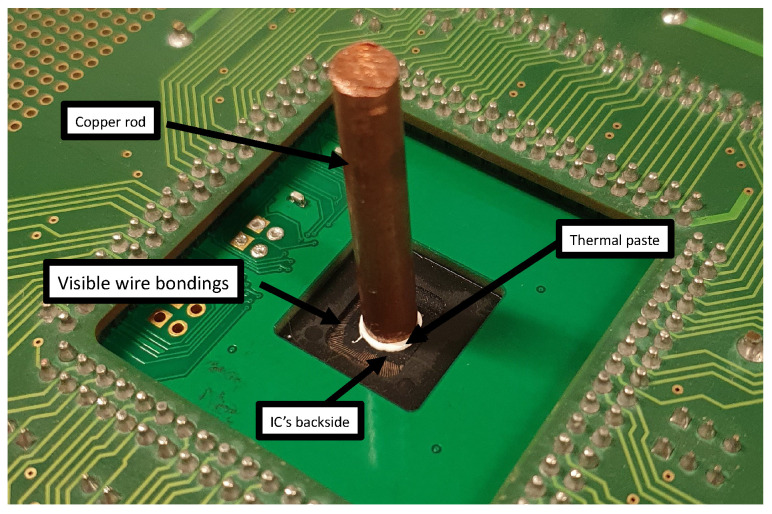
An attempt at restoring the thermal behavior of the IC with a copper rod.

If one has access to an electronics lab, trying to bypass the verification protocol with a removable heat sink is a natural solution. To try to restore the initial thermal behavior of our six ICs with a backside opening, we decided to use a copper rod (fitting into the cavity created to obtain access to the backside) as a heat sink and thermal paste, as shown in Figure 7. The approach was purely empirical because we had no knowledge of how the thermal dissipation capability of each IC was altered.

As discussed above, it is difficult to predict the thermal dissipation capability of the IC after opening the backside. Indeed, the thermal resistance of the tampered package is unknown. As a result, we first selected a 27 mm long rod in an attempt to bypass the verification protocol by restoring the initial thermal behavior of the IC in its intact package. Because this attempt was unsuccessful, we cut the rod into two pieces: one 9 mm long and the other 18 mm long. This variation in length was intended to affect the thermal dissipation capability of the system constituted by IC and the rod. Indeed, according to Newton’s law of cooling, the convection heat loss ($Q_{conv}$), the primary mode of heat transfer from the rod to the surrounding air, can be expressed as follows:

(4) $$Q_{conv}=h\cdot A\cdot \left(T_{rod}-T_{air}\right)$$

with A=π·d·L, the surface area of the rod, which is proportional to the rod’s diameter (d) and length (L). Therefore, dividing *L* by 1.5 and 3 allowed us to significantly change the convection loss of the system.

After placing a rod on the backside of each IC, the values $\bar{\beta_{1}}$ and $\sigma_{\beta_{1}}$ were collected as in previous experiments. Table 2 presents the results obtained for the three rods used and recalls the values obtained when the package was intact. It can be observed that, with the 27 mm long rod, the $\bar{\beta_{1}^{27}}+3\cdot \sigma_{\beta_{1}^{27}}$ values for all ICs are significantly below the corresponding $\bar{\beta_{1}}-3\cdot \sigma_{\beta_{1}}$ values. As a result, the 27 mm long heatsink dissipates more heat than the original package, and the package verification protocol would indicate tampering with the back of the package.

Assuming a normal distribution of $\beta_{1}$ values, we can calculate the probability that tampering will not be detected by the verification protocol. In the case of the 27 mm rod, this probability is zero, as shown in Table 3, except for the IC labeled AA, for which the probability is about 7 %. For shorter rods, the probabilities are higher, and the 9 mm long rod appears to be the best choice for an adversary attempting to bypass the verification protocol. It should be noted, however, that it is unlikely to find a rod that is perfectly adapted to all ICs with a backside opening. In fact, the 18 mm rod provides a greater chance of bypassing the verification protocol for devices marked AA and AF, and a shorter rod could have yielded better results for devices AE and AG. This can likely be explained by process variations in IC manufacturing, as well as variations in the IC opening process, which requires mechanical polishing. The manual application of thermal paste to ensure good thermal contact between the substrate and the wafer also has an effect. Overall, we may conclude that using a removable heatsink appears to be a risky way for an adversary to bypass the verification protocol.

#### 4.3.2. IC Pre-Heating

Removing the back of the package results in a sharper linear increase in the internal IC temperature at power-up and, thus, in the $\beta_{1}$ values. An adversary attempting to bypass the verification protocol should find a way to force the $\beta_{1}$ value back to some unknown acceptable range. One possible solution for an adversary might be to break the linear rise in temperature at power-up to fool the linear regression analysis and obtain a lower $\beta_{1}$. For this, they could use an air gun to raise the internal temperature of the IC above the ambient temperature just before powering up. In fact, this could allow them to force the IC to first experience a temperature decrease followed by a temperature increase during the 300 ms that the IC spends checking the integrity of the package, thus breaking the linear trend and fooling the linear regression analysis. This could force the IC to compute a lower $\beta_{1}$ value that, with luck, might fall within the acceptable range.

**Figure 8:**
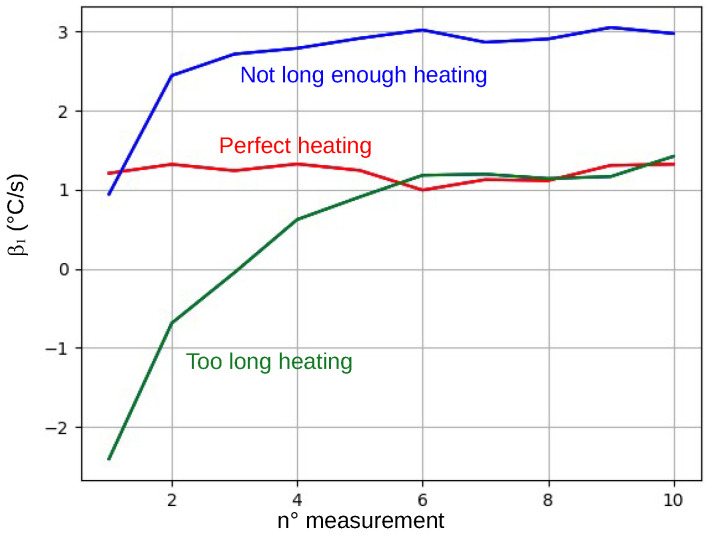
The three possible evolutions of the 10 successive $\beta_{1}$ values when preheating the IC with an air gun.

We thus intended to bypass the implemented verification protocol using this approach. During these trials, the ICs were programmed to calculate ten $\beta_{1}$ in a row. After about ten trials, we succeeded in bypassing the verification protocol by analyzing the evolution of $\beta_{1}$ values to determine whether we heated the IC too much or too little before powering it up, an analysis that is not possible for an adversary. Figure 8 shows the three different evolutions that we observed for the successive values of $\beta_{1}$ during these trials. As expected, if the internal temperature of the IC is initially forced to too high a value, the obtained $\beta_{1}$ values will be negative (green curve in the figure), indicating that the IC is cooling down despite being powered up. In this case, the verification protocol detects the tampering of its backside package. Similarly, if the internal temperature is initially raised to too low of a value, the effect on the linear regression analysis will not be sufficient to force $\beta_{1}$ back into the acceptable range (blue curve), and the verification log will flag a package change. Finally, with luck, it is possible to preheat the IC to an appropriate value and bypass the verification protocol. Of course, the probability to properly preheat the IC depends on the width of the acceptable $\beta_{1}$ values.

## 5. Discussion: Impact of the Temperature Sensor

Of course, the likelihood of bypassing the package integrity verification protocol depends on the width of the acceptable $\beta_{1}$ values. For our protocol, this seems dependent on the accuracy of the temperature sensor, which is limited to ±1.5 °C for our device under test. It is not uncommon for microcontrollers to include temperature sensors with better accuracy. To estimate the benefits of using a more accurate temperature sensor, we emulated one by repeating and averaging (off-board) the sequence of 25 measurements provided by our DUT and considered in the previous experiments. Averaged values were then used to compute the $\bar{\beta_{1}}$ and $\sigma_{\beta_{1}}$ values.

**Figure 9:**
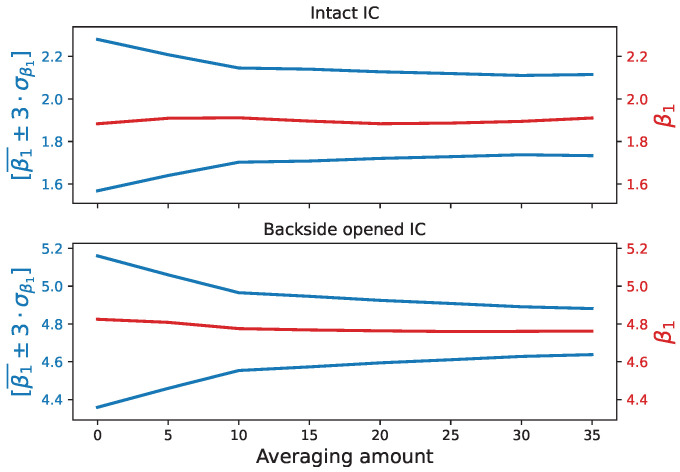
Evolution of the interval $\left[\beta_{1}\pm 3\cdot \sigma_{\beta_{1}}\right]$ and the average $\beta_{1}$ value as we increase the number of measurements.

Figure 9 shows the trend with *n* of the acceptable range $\bar{\beta_{1}}\pm 3\cdot \sigma_{\beta_{1}}$ for two ICs, one with a backside-tampered package and one with an intact package. As expected, the acceptable range decreases with *n* according to a square root law, while $\bar{\beta_{1}}$ remains stable. Thus, the use of a more accurate sensor should render the bypass of the verification protocol harder. As an illustration, following the theoretical square root decay of $\sigma_{\beta_{1}}$ with *n*, Table 4 predicts the probability of bypassing the verification protocol using the copper rod, as tested experimentally in the previous section (see Figure 7).

The comparison with Table 3 shows that the number of cases with a null probability of bypassing the verification is greater. In other words, it is harder to find the appropriate removable heatsink. Furthermore, when the probabilities are not zero, they are lower due to the reduction of the overlapping range of the probability density function (PDF), explained by the reduction of $\sigma_{\beta_{1}}$. This reduction has a double effect on the probabilities to bypass the verification protocol: first, it reduces the acceptable range $\left[\bar{\beta_{1}}\pm 3\cdot \sigma_{\beta_{1}}\right]$ of the IC in an intact package, and, second, it reduces the range of possible $\bar{\beta_{1}}$ values observed for an IC with a tampered package. Therefore, there is a double benefit to using a more accurate temperature sensor.

## 6. Conclusions

Security considerations have become fundamental in modern integrated circuit design, particularly due to the increasing demand for secure applications across various domains. This includes vulnerable Internet of Things devices, secure Smartcards, and critical cyber-physical systems.

As IC packaging varies significantly between applications, and nearly all ICs require packaging, ensuring package integrity to defend against physical attacks is a crucial, yet often overlooked, area of study.

The result suggest that proactive package integrity verification, leveraging either specialized or commonly available embedded temperature sensors, represent a promising approach to bolster the security of IoT devices and other vulnerable systems.

Our analysis revealed vulnerabilities in the proposed countermeasure. While most attempted attacks were impractical, placing heat sinks on the backside could allow a bypass of the system. However, this method is difficult to implement due to variations in IC and package manufacturing processes, as well as the package opening process itself.

Further research is needed to confirm these findings and to determine the effects of opening size and position, as well as temperature sensor number and placement. Additional research should also explore the feasibility of detecting frontside package tampering using more accurate sensors. Ultimately, proactive package integrity verification is becoming increasingly vital for securing the expanding landscape of integrated circuit applications.

## Figures and Tables

**Table 1:** The following table presents the means and standard deviations of $\bar{\beta_{1}}$ and $\sigma_{\beta_{1}}$ obtained after 25 power-up cycles, both before and after the backside package was opened.

IC id	Intact Package		Backside Opening		
	$\bar{\beta_{1}}$	$\sigma_{\beta_{1}}$	$\bar{\beta_{1}^{\prime }}$	$\sigma_{\beta_{1}^{\prime }}$	$\bar{\beta_{1}}-\bar{\beta_{1}^{\prime }}$
AA	1.400	0.125	7.470	0.063	6.070
AB	1.608	0.147	5.889	0.089	4.291
AC	1.636	0.112	5.642	0.068	4.006
AG	2.095	0.195	4.097	0.077	2.002
AE	2.970	0.175	5.817	0.084	2.847
AF	3.101	0.453	5.660	0.059	2.559

**Table 2:** Average values and standard deviation of 25 measurements of $\beta_{1}$ for the same IC batch with three distinct heatsinks. Units are expressed in °C/s.

IC id	Intact Package		9 mm HS		18 mm HS		27 mm HS	
	$\bar{\beta_{1}}$	$\sigma_{\beta_{1}}$	$\bar{\beta_{1}^{9}}$	$\sigma_{\beta_{1}^{9}}$	$\bar{\beta_{1}^{18}}$	$\sigma_{\beta_{1}^{18}}$	$\bar{\beta_{1}^{27}}$	$\sigma_{\beta_{1}^{27}}$
AE	2.970	0.175	1.670	0.318	0.849	0.223	0.709	0.047
AB	1.608	0.147	1.515	0.257	0.812	0.113	0.735	0.047
AC	1.636	0.112	1.638	0.288	0.909	0.145	0.708	0.109
AG	2.095	0.195	1.143	0.204	0.660	0.148	0.516	0.073
AA	1.400	0.125	1.762	0.211	0.941	0.122	0.816	0.142
AF	3.101	0.435	1.455	0.074	0.890	0.177	0.714	0.095

**Table 3:** Probabilities (calculated assuming a normal distribution of $\beta_{1}$) to bypass the verification protocol using a removable heatsink.

IC id	Intact Package $\left[\bar{\beta_{1}}\pm 3\cdot \sigma_{\beta_{1}}\right]$	9 mm HS Probability	18 mm HS Probability	27 mm HS Probability
AE	[2.445, 3.495]	0.740 %	0.000%	0.000%
AB	[1.167, 2.049]	91.215%	0.084%	0.000%
AC	[1.300, 1.972]	87.692%	0.350%	0.000%
AG	[1.510, 2.680]	3.601%	0.000%	0.000%
AA	[1.025, 1.775]	52.456%	24.556%	7.053%
AF	[1.742, 4.460]	0.000%	0.000%	0.000%

**Table 4:** Predicted probabilities to bypass the verification protocol using a removable heatsink in case of a temperature sensor with an accuracy about ±0.45 °C instead of ±1.5 °C.

IC id	9 mm HS Probability	18 mm HS Probability	27 mm HS Probability
AE	0.000%	0.000%	0.000%
AB	69.35%	0.000%	0.000%
AC	75.66%	0.000%	0.000%
AG	0.000%	0.000%	0.000%
AA	0.000%	0.000%	0.000%
AF	0.000%	0.000%	0.000%

## Data Availability

The datasets generated during and/or analyzed during the current study are available from the corresponding author on reasonable request.
